# Cellular Response of Adapted and Non-Adapted *Tetrahymena thermophila* Strains to Europium Eu(III) Compounds

**DOI:** 10.3390/biology13050285

**Published:** 2024-04-23

**Authors:** Patricia Alonso, Javier Blas, Francisco Amaro, Patricia de Francisco, Ana Martín-González, Juan Carlos Gutiérrez

**Affiliations:** Department of Genetics, Physiology and Microbiology, Faculty of Biology, Complutense University of Madrid, 28040 Madrid, Spain; patrialan22@gmail.com (P.A.); jblas02@ucm.es (J.B.); pamaro@bio.ucm.es (F.A.); pafranci@ucm.es (P.d.F.); anamarti@ucm.es (A.M.-G.)

**Keywords:** europium, lipid metabolism, bioaccumulation, oxidative stress, gene expression, *Tetrahymena thermophila*

## Abstract

**Simple Summary:**

The analysis of the toxicity of lanthanides, and among them europium, has grown in recent years due to its multiple applications in different human technologies. In the present research work, we analyze its toxicity in the eukaryotic microorganism model *Tetrahymena thermophila*, comparing strains adapted to high concentrations of two europium compounds (chloride and oxide forms) with the wild-type strain. The oxidative stress caused by europium oxide is reduced by overexpression of genes encoding various antioxidant enzymes. Similarly, metallothionein genes of this microorganism are overexpressed, which could indicate the possible chelation of this lanthanide by these proteins. Lipid metabolism and autophagy are involved in the cellular stress response to europium. Both bioaccumulation in vacuoles, and their subsequent release, as well as a possible biotransformation to europium phosphate are involved in the europium detoxification process in these cells. A better understanding of the cellular mechanisms of lanthanide detoxification is very useful information for solving bioremediation problems and possible intoxications in animals and humans.

**Abstract:**

Europium is one of the most reactive lanthanides and humans use it in many different applications, but we still know little about its potential toxicity and cellular response to its exposure. Two strains of the eukaryotic microorganism model *Tetrahymena thermophila* were adapted to high concentrations of two Eu(III) compounds (EuCl_3_ or Eu_2_O_3_) and compared to a control strain and cultures treated with both compounds. In this ciliate, EuCl_3_ is more toxic than Eu_2_O_3_. LC_50_ values show that this microorganism is more resistant to these Eu(III) compounds than other microorganisms. Oxidative stress originated mainly by Eu_2_O_3_ is minimized by overexpression of genes encoding important antioxidant enzymes. The overexpression of metallothionein genes under treatment with Eu(III) compounds supports the possibility that this lanthanide may interact with the -SH groups of the cysteine residues from metallothioneins and/or displace essential cations of these proteins during their homeostatic function. Both lipid metabolism (lipid droplets fusing with europium-containing vacuoles) and autophagy are involved in the cellular response to europium stress. Bioaccumulation, together with a possible biomineralization to europium phosphate, seems to be the main mechanism of Eu(III) detoxification in these cells.

## 1. Introduction

In the last decade, there has been growing interest in the potential toxicity of lanthanides (Ln), due to their extensive human use in many new technologies [1,2,3,4,5]. Some of which are: electronic devices (television screens, computers, cell phones, etc.), manufacturing (ceramic pigments, colorants in glassware, etc.)) and renewable energies (solar cells, hybrid automobiles and biofuels catalysis) [6,7]. In addition, they have a special interest in biomedical applications, for instance: nuclear medicine imaging, magnetic resonance, and as fluorescent probes in optical cellular imaging [6,8,9,10]. Ln have also been used as fertilizers in oriental agriculture. Approximately 50 to 100 tons of Ln oxides have been used annually in Chinese agriculture [11]. Moreover, because of agricultural and industrial activity, Ln have been detected in wastewater and aquatic ecosystems. Likewise, Ln has been used as a feed supplement to promote the growth of farm animals, which has led to the presence of Ln in soil through animal wastes [2,12,13]. Several studies indicate the potential health risks for humans from this Ln pollution by two main ways, directly by occupational exposure and indirectly through ingestion of water or food containing these pollutants due to agriculture [5,14,15,16]. 

In the periodic table, the lanthanides group consists of 15 metals, which together with yttrium (Y) and scandium (Sc), form the so-called rare earth elements (REEs). The term “rare” does not mean that they are scarce in the Earth’s crust, for example, cerium (Ce) is about 100 times more abundant than cadmium (Cd), one of the most toxic metals. Despite the significant risks for ecosystems and/or organisms, as stated by many authors, ecotoxicological studies to assess the REE toxicity of these emerging pollutants are still scarce. Many of these studies focus exclusively on a few REEs, particularly gadolinium (Gd), lanthanum (Ln), and cerium (Ce) [1,4,12,15,17]. Most of them mainly analyze their effects on cell growth and viability, with a lower knowledge on molecular, physiological, and cellular structural effects.

Europium (Eu) is a relatively rare Ln in the Earth’s crust (an average of 2–2.2 ppm), that is not found as a free element but as a component of many minerals. Like other Ln its predominant oxidation state is +3, but it can also readily form divalent (+2) compounds, which is unusual for other lanthanides. Eu is the most reactive of the REEs [12]. It is one of the chemical elements that form fluorescent compounds that are used in devices such as color televisions, fluorescent lamps, LEDs (light-emitting diodes), LCD screens and many lighting systems. Europium oxide (Eu_2_O_3_) is widely used as a fluorescent element in television sets [7,8,9,18,19]. Other notable uses of Eu include: (a) the European Union uses Eu in the ink of euro bills, to prevent counterfeiting. Depending on the molecule of which it is a part, Eu can emit red, green, or blue light. Under special laser or ultraviolet (UV) light, the outline of Europe on the bills shines greenish, the crown of stars is yellow or red, and the monuments, signatures and hidden seals are dark blue. An example is shown in the graphical abstract (an image of a €20 bill under UV light), (b) in astrophysics, the Eu signature from the light spectrum emitted by a star can be used to classify stars [20].

Compared to other metals, Eu does not exhibit significant toxicity in both its chloride and oxide forms, although it is slightly more toxic in its nitrate form [16]. Its toxicity as the EuCl_3_ salt is comparable to other REEs, and it has been shown to be quite harmful during the early life stages to sea urchins [21]. Toxicity of Eu hydroxide nanoparticles in mice is low even at high concentrations [22]. For humans, prolonged exposure to Eu vapors can be very dangerous, causing pulmonary embolism, and it can be a danger to the liver if it accumulates in the body [5]. 

Due to the increasing demand for rare earth metals by modern human technologies, their possible bioaccumulation by microorganisms, such as bacteria, has been studied [23]. The thermophilic bacterium *Thermus scotoductus* has shown a high capacity to tolerate high Eu concentrations, to biomineralize it and to bioaccumulate it intra- and extracellularly [24]. Likewise, biosorption has been considered as an inexpensive and environmentally friendly mechanism to recover REEs from industrial electronic waste [25]. Good results have been obtained in studies to recover Eu(III) and other Ln using microalgae [26], bacteria [27,28], filamentous fungi [29] or yeasts [30]. The ciliate protozoan *Paramecium* sp. can transform aqueous inorganic forms of Eu(III), Pb(II) or U(VI) into organic complexes by binding to glycoproteins present in the ciliate coat, initiating this process as a biosorption mechanism [31]. 

*Tetrahymena thermophila* is a eukaryotic microorganism (ciliate-model) widely used in ecotoxicological studies [32,33,34,35,36,37,38], because it has an animal biology and more human orthologous genes than other eukaryotic microorganisms (such as yeasts). Among 874 human orthologous genes present in *T. thermophila*, 58 are related to human diseases [39]. Therefore, *T. thermophila* is an excellent eukaryotic model for comparative ecotoxicological analysis with higher organisms, including humans. 

In the present study, two strains of the ciliate *T. thermophila* adapted to high concentrations of EuCl_3_ or Eu_2_O_3_ were obtained and compared with the control strain. The toxicity levels of both Eu(III) compounds, and their effects on ciliate growth, have been studied. An analysis of the oxidative stress induction by these Eu compounds was carried out. The bioaccumulation capacity of Eu by these adapted strains was also studied by transmission electron microscopy (TEM) and microanalysis (TEM-XEDS). In addition, the expression of nine different genes encoding antioxidant enzymes, together with four general stress genes (encoding metallothioneins) were analyzed by qRT-PCR in both Eu-adapted strains. All these analyses together give us a general overview of the cellular stress response to Eu in this eukaryotic microorganism.

## 2. Materials and Methods

### 2.1. Microorganism, Culture Conditions and Eu-Adapted Strains

Dr. E. Orias (University of California, USA), kindly supplied the *Tetrahymena thermophila* strain SB1969. This ciliate was axenically grown in PP210 medium (as described in [40]), and cell cultures were maintained at a constant temperature of 30 ± 1 °C. The Eu(III) compounds used were: europium chloride (EuCl_3_) (Acrosorganics) and europium oxide (Eu_2_O_3_) (Aldrich Chemistry).

In all experiments with *T. thermophila* growing in PP210 medium, aqueous solutions of EuCl_3_ (2 mM) were used, while Eu_2_O_3_ (4 mM) was used in aqueous solution containing 53.6% 1 N HCl. Both concentrations correspond to approximately half of the LC_50_ values obtained for each Eu compound. Experiments performed in Tris-HCl buffer were carried out from a culture previously grown (48 h or late exponential phase) in PP210, and subsequently resuspended in 0.01 M Tris-HCl buffer (pH 7.5), after centrifugation (2400 rpm, 2 min) of the culture grown in PP210.

The adaptation process to EuCl_3_ or Eu_2_O_3_, which lasted about 8 months, consisted of gradually exposing strain SB1969 to increasing concentrations of each Eu compound, until the maximum tolerated concentration (MTC) was reached. In this way, cells adapted to a very high EuCl_3_ (5.5 mM) (TtEuCl_3_-adap strain) or Eu_2_O_3_ (8.5 mM) (TtEu_2_O_3_-adap strain) concentrations were selected and both strains were maintained over time in PP210 medium in the constant presence of the Eu(III) compounds at the corresponding MTC.

### 2.2. Growth Kinetics and LC_50_ Calculation by Flow Cytometry

Growth curves were obtained for the three strains (control, TtEuCl_3_-adap and TtEu_2_O_3_-adap) by counting cells in a Neubauer chamber from aliquots of the different cultures at different times. Subsequently, using the informatics application DMFit (http://browser.combase.cc/DMFit.aspx, accessed on 21 September 2023), growth curves were constructed according to the model proposed by [41]. The two parameters obtained from the growth curves were the specific growth rate (or speed) μ (h^−1^) and the generation time Tg (h).

Flow cytometry was used to calculate cell mortality and LC_50_ values for Eu treatments. Samples were prepared from control cultures (100 mL) after reaching the end of the exponential phase (1–3 − 10^5^ cells/mL). Cells were centrifuged (2400 rpm, 2 min) and washed twice with 0.01 M Tris-HCl buffer (pH 7.5). Two of the samples were resuspended in TrisHCl buffer and two others in PP210 medium. All these samples were treated with increasing concentrations of europium (EuCl_3_ or Eu_2_O_3_), at 30 °C for 24 h. Each test was repeated three times to corroborate the results. LC_50_ values were estimated by using Probit analysis with Statgraphics Centurion XVI and STATA 9 (confidence 95%, *p* < 0.05) as described in [42]. Cell mortality was estimated by adding to the cell suspension the fluorophore propidium iodide (PI) (Sigma, Kawasaki, Japan) at a final concentration of 2.5 μg/mL. PI only penetrates and stains membrane-damaged cells (dead or severely damaged cells) [43]. Therefore, non-viable cells can be identified as PI-positive and quantified by fluorescence at 670 nm Lp (long pass) in the FL3 channel. The flow cytometer used was FACScalibur (Becton & Dickinson, Franklin Lakes, NJ, USA), equipped with an argon-ion excitation laser (488 nm). Three types of controls were used: (1) blank-sample: cells not exposed to metal and without PI, which allows for calibrating the cytometer and detecting autofluorescence from the sample. (2) negative or live control: cells not exposed to metal but treated with PI, to evaluate basal mortality, and (3) positive or dead control: cells fixed with formaldehyde (37%) and treated with PI, to check the proper functioning of the cytometer.

### 2.3. Oxidative Stress Detection

To elucidate whether Eu compounds induce cellular production of peroxide radicals, the fluorochrome 2′-7′dichlorodihydrofluorescein diacetate (DA-DCDH2F) (Sigma) was used; this compound is incorporated into cells and subsequently, by the action of esterases, is converted to DCDH2F (which is non-fluorescent). However, if free radicals are present, it is rapidly oxidized to 2′-7′dichlorofluorescein (DCF) which is highly fluorescent (absorbs at a wavelength of approximately 485–500 nm and emits between 515–530 nm). This molecule is quite specific for peroxides (including hydrogen peroxide), peroxinitrite and hydroxyl radical detection, but cannot detect superoxide anions [44]. In addition to the cultures treated with the Eu compounds, a positive control was performed by exposing the cells to the oxidizing agent menadione (MD) (Sigma) at a final concentration 2 mM for 30 min [45]. Fluorescence emission was quantified by flow cytometry.

### 2.4. Transmission Electron Microscopy (TEM) and Microanalysis (TEM-XEDS)

For ultrastructural analysis (TEM), both Eu-adapted strains (TtEuCl_3_-adap and TtEu_2_O_3_-adap), a culture treated (1 or 24 h) with EuCl_3_ (2 mM), and a control (untreated) culture were chosen. All cellular samples were processed according to the protocol described in [46]. Briefly, after fixation and dehydration, the samples were embedded in Embed 812 resin (TAAB), following the manufacturer’s instructions. The ultrathin sections, after contrasting with uranyl acetate (2% in distilled water) and Reynolds solution (lead citrate), were observed in a JEM 1010 (JEOL) transmission electron microscope at 80 Kv, and the images were captured with a Megaview II camera.

To analyze the elemental composition of the vacuolar electrodense deposits observed by TEM the TtEuCl_3_-adapted strain was selected, and a microanalysis was performed using TEM-XEDS. Cells were fixed with glutaraldehyde (2.5%), washed with cacodylate buffer, dehydrated, and embedded in resin similar to the protocol used for TEM. Thin sections (1 μm) were observed under the JEM 2100HT electron microscope (JEOL) at 200 Kv, which incorporates an XEDS (X-ray Energy Dispersive Spectroscopy) microanalysis system (Oxford INCA, Oxford, UK).

### 2.5. Total RNA Isolation and cDNA Synthesis

Exponential cell cultures (1–3 × 10^5^ cells/mL) from control, EuCl_3_ treated (24 h) culture and Eu-adapted cultures (constantly exposed to Eu compounds at each corresponding MTC) were harvested by centrifugation at 2400 rpm for 2 min. Total RNA samples were isolated by using the TRIzol Reagent^®^ method (Molecular Research Center, Inc., Cincinnati, OH, USA). RNA samples were treated with DNase I (Roche) for 30 min at 37 °C. RNA integrity was tested by agarose (1.2%) gel electrophoresis and sample concentrations were calculated spectrophotometrically by the NanoDrop 1000 (Thermo Scientific, Waltham, MA, USA). First strand cDNA synthesis from total RNA (3 μg) was carried out using the commercial 1st Strand cDNA Synthesis kit for RT-PCR (AMV) (Roche, Basel, Switzerland) and oligo(dT)-adaptor primer (Roche). The retrotranscription reactions were performed in a Mastercycler gradient thermocycler (Eppendorf, Hamburg, Germany), following this temperature program: 10 min at 25 °C, 60 min at 42 °C, and 5 min at 99 °C. 

### 2.6. Quantitative RT-PCR (qRT-PCR)

cDNA samples were amplified in duplicated in 96-well microtiter plates. Each qRT-PCR reaction (20 μL) contained: 2 μL of a 10-1 dilution of the corresponding cDNA sample and 18 μL of a master mix, consisting of SYBR Green 1x (Takara, San Jose, CA, USA), the Rox 1x dye (Takara), which is used to normalize fluorescence intensity, sterile water and the previously designed primer pair for each selected gene (0.2 μM). PCR primers (Table S1) were designed using the “Primer Quest and Probe Design” online application from IDT (Integrated DNA Technologies, https://eu.idtdna.com/PrimerQuest/Home/Index, accessed on 12 May 2023), and synthetized by the commercial companies Invitrogen or IDT. Beta-actin gene (*TtACT*) was used as an endogenous control or normalizer gene, as it has been validated as a reference gene in qRT-PCR under either biotic or abiotic stresses [47]. Melting curves were obtained and primers specificity was tested by confirming each PCR product (amplicon) by gel electrophoresis and sequencing. Real-time PCR reactions were carried out in an iQ5 real-time PCR apparatus (Bio-Rad, Hercules, CA, USA) and the thermal cycling protocol was as follows: 5 min at 95 °C, 40 cycles (30 s at 95 °C, 30 s at 55 °C, and 20 s at 72 °C), 1 min at 95 °C, and 1 min at 55 °C. All controls (no template controls (NTC) and RT minus controls) were negative. Amplification efficiency (E) was measured by using 10-fold serial dilutions of a positive control PCR template. qRT-PCR efficiency parameters (corroborated in quadruplicate, with intra- and inter-plate replicates) were obtained for each gene (Table S2). Finally, the results were processed by the standard curve method [48]. 

### 2.7. Statistical Analysis

The dose-response graphics, its mathematical function and LC_50_ values were calculated using the software package Stratgraphics Centurion 16.0. Data could be adjusted to a dosage-mortality sigmoid curve using the Probit model, and then the LC_50_ was estimated. Gene expression differences (qRT-PCR analysis) were tested for statistical significance by one-way ANOVA followed by a Dunnett’s multiple comparisons test performed with GrapPad Prism 10.1.0 (316). *p*-value was fixed at ≤0.05 for statistically significant values and ≤0.01, 0.001, or 0.0001 for high or very high significance values.

## 3. Results

### 3.1. Ecotoxicological Parameters 

**Figure 1:**
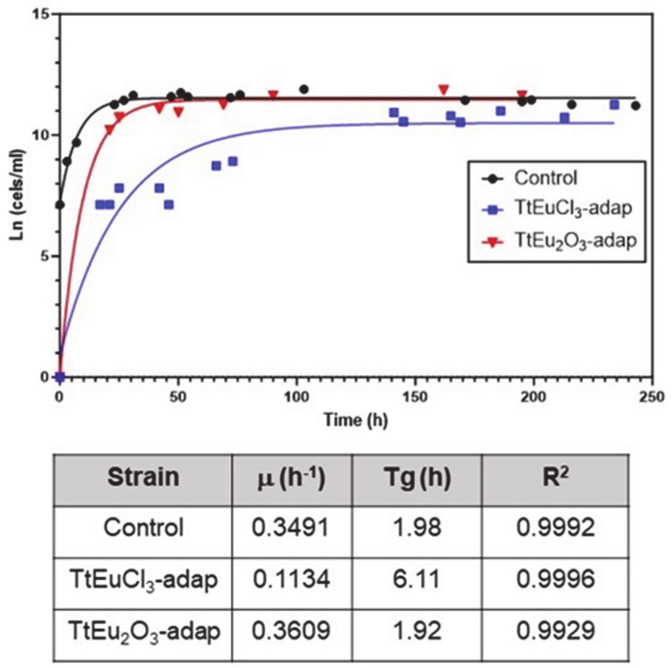
Growth curves of the two adapted strains and the control. Below are the growth parameters: growth rate (μ), generation time (Tg) and correlation coefficient (R^2^).

LC_50_ values for both Eu(III) compounds were obtained from dose–mortality curves (Figure S1) of T. thermophila cultures grown in PP210 medium or maintained in 0.01 M Tris-HCl buffer. These values are listed in Table 1. The growth curves and the parameters derived from them are shown in Figure 1. Comparative analysis of the growth curve parameters of the strains adapted to Eu(III) compounds with regard to the untreated control shows that the TtEuCl_3_-adap strain has a growth rate about three times lower than the control or the TtEu_2_O_3_-adap strains, and a generation time (Tg) about three times longer (Figure 1). In contrast, the TtEu_2_O_3_-adap strain has a growth rate and Tg very similar to the control strain (Figure 1).

### 3.2. Oxidative Stress Assessment

**Figure 2:**
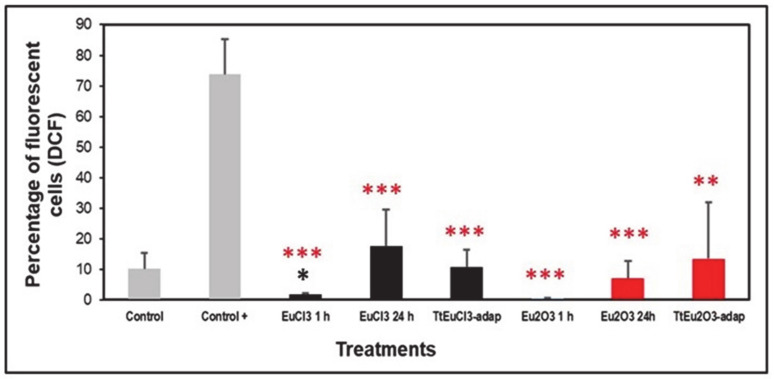
Peroxide generation detected by DCF fluorescence. Gray bars: control (untreated) and positive control (+). Black bars: EuCl_3_-treated cultures (1 or 24 h) and TtEuCl_3_-adapted strain. Red bars: Eu_2_O_3_-treated cultures (1 or 24 h) and TtEu_2_O_3_-adapted strain. Red stars represent significant differences with respect to the positive control and the black star represents significant differences with respect to the untreated control. Stars denote significant differences [*p* ≤ 0.05 (*), *p* ≤ 0.01 (**), *p* ≤ 0.001 (***)].

The level of oxidative stress was assessed based on the generation of peroxides measured by DCF fluorescence. Figure 2 shows the results expressed as a percentage of fluorescent cells (DCF positive) compared to two controls (an untreated control and a positive control treated with the oxidizing agent MD). All europium-treated (1 or 24 h) and the adapted strains (TtEuCl_3_-adap and TtEu_2_O_3_-adap) showed significantly lower values (*p* ≤ 0.001, and 0.01, respectively) than the positive control, and only the EuCl_3_ 1 h sample was significantly lower (*p* ≤ 0.05) than the untreated control (Figure 2). All europium-treated or europium-adapted cultures showed similar or lower percentages of fluorescent cells than the untreated control, indicating that Eu(III) does not cause significant levels of oxidative stress (peroxide generation) in this ciliate. 

### 3.3. Comparative Quantitative Expression Analysis of Several Genes Involved in Oxidative and/or General Stress

Thirteen genes involved in general cellular stress or in counteracting oxidative stress were selected for this analysis, based on our previous studies. They are: one glutathione reductase (*GR1*), two paralogous thioredoxin reductase genes (*TrxR2*, *TrxR5*), the only catalase (*CAT*) and glutathione cysteine ligase (*GCL*) genes present in this ciliate genome, two superoxide dismutases (*Fe-SOD*, *Cu-SOD*), two glutathione S-transferases (*GSTM3*, *GSTZ2*) and the five metallothionein genes (*MTT1*, *MTT2/4*, *MTT3*, *MTT5*) present in this *Tetrahymena* species. qRT-PCR analysis was carried out on cultures of *T. thermophila* treated with EuCl_3_ (2 mM) or Eu_2_O_3_ (4 mM) for 24 h, and on both adapted strains (TtEuCl_3_-adap and TtEu_2_O_3_-adap). The *MTT2/4* gene pair has a high identity between them (98%) [49], so it is not possible to design primers that can discriminate between them, and for this reason the obtained gene expression values probably represent the sum of both genes. 

**Figure 3:**
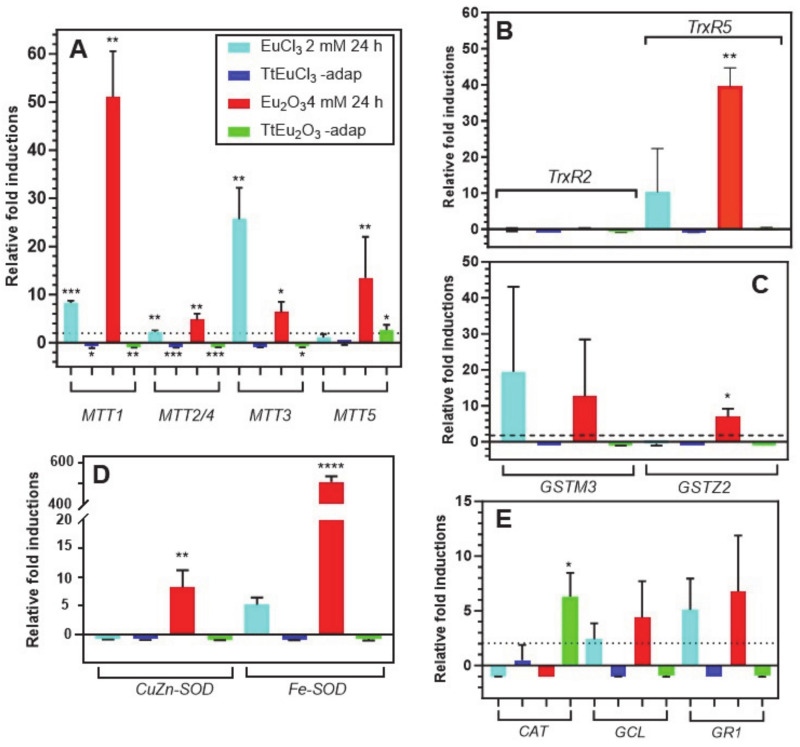
Stress gene expression analysis by qRT-PCR. (**A**): metalothioneins (*MTT*). (**B**): thioredoxin reductases (*TrxR*). (**C**): glutathione S-transferases (*GST*). (**D**): superoxide dismutases (*SOD*). (**E**): catalase (*CAT*), glutathione cysteine ligase (*GCL*) and glutathione reductase (*GR*). Stars denote significant differences [*p* ≤ 0.05 (*), *p* ≤ 0.01 (**), *p* ≤ 0.001 (***), *p* ≤ 0.0001 (****)]. A gene expression induction is considered positive when the fold-induction value obtained is >2 (indicated by the dashed line).

Figure 3A shows the results obtained for the five metallothionein isoforms present in this ciliate. The *MTT1* gene is significantly overexpressed under the stress induced by EuCl_3_ (~8-fold) and Eu_2_O_3_ (~51-fold). By contrast, this gene is significantly repressed in both adapted strains. This MT gene is the most overexpressed under Eu_2_O_3_ (Figure 3A). The *MTT2/4* pair behaves similarly to *MTT1*, although with much lower induction levels under both EuCl_3_ and Eu_2_O_3_ treatments. Note the significant (*p* ≤ 0.01) induction (~4.8-fold) under Eu_2_O_3_ stress. The *MTT3* gene, unlike the other MT genes, is most highly overexpressed under the presence of EuCl_3_ (~25-fold), compared to the Eu_2_O_3_ stress (~6-fold), and like the previous ones, it is repressed in both adapted strains (Figure 3A). Finally, the *MTT5* gene is significantly overexpressed (~13-fold) almost exclusively under treatment with Eu_2_O_3_, and unlike the rest of the MT genes, this one shows some significant expression level (~2.6-fold) in the Eu_2_O_3_-adapted strain (Figure 3A). 

The two selected *TrxR* genes show very different expression results (Figure 3B), under stress by europium compounds. There is no expression of the *TrxR2* gene under treatment with europium compounds or in both europium-adapted strains. In contrast, *TrxR5* gene expression is induced in cultures treated with EuCl_3_ (~8.5-fold) and strongly and significantly with Eu_2_O_3_ (~39.5-fold) (Figure 3B). Similarly, the expression induction patterns of the two selected *GST* genes are very different (Figure 3C). The *GSTM3* gene is induced under stress by EuCl_3_ (~19-fold) and with Eu_2_O_3_ (~12.6-fold), both values with very large standard deviations. This could be the reason for the lack of a significant average value with respect to the control gene. In contrast, the *GSTZ2* gene is only significantly induced under Eu_2_O_3_ stress (~7-fold), while there is no induction in the adapted strains (Figure 3C).

The expression levels of the two *SOD*-encoding genes are shown in Figure 3D. The *CuZn-SOD* gene is induced (~6.7-fold) exclusively under Eu_2_O_3_ treatment. However, the *Fe-SOD* gene is induced under treatment with both europium compounds: about 5-fold with EuCl_3_ and about 500-fold with Eu_2_O_3_, this average value being highly significant (*p* ≤ 0.0001) with respect to the control gene. In both adapted strains, no expression of these genes is detected, but rather a certain repression (Figure 3D).

The expression results of the last three selected genes (*CAT*, *GCL* and *GR1*) are shown in Figure 3E. The catalase (*CAT*) gene is repressed in the EuCl_3_- and Eu_2_O_3_-treated cultures, but some significant induction (~6-fold) occurs in the TtEu_2_O_3_-adap strain. The other two genes (*GCL* and *GR1*) show a very similar gene expression induction pattern, with expression only under EuCl_3_ and Eu_2_O_3_ treatments, but with average values with high standard deviations. The two adapted strains show no expression of these two genes, but there is some repression (Figure 3E). 

### 3.4. Ultrastructural Analysis

**Figure 4:**
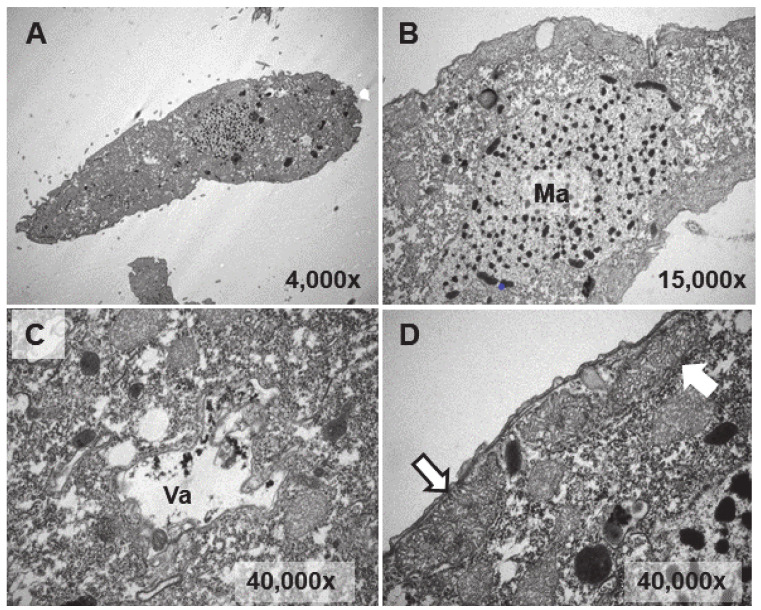
TEM images of cells from a control culture (untreated). (**A**): *T. thermophila* whole cell. (**B**): Macronucleus (Ma). (**C**): Vacuole (Va). (**D**): Mitochondria (arrows).

**Figure 5:**
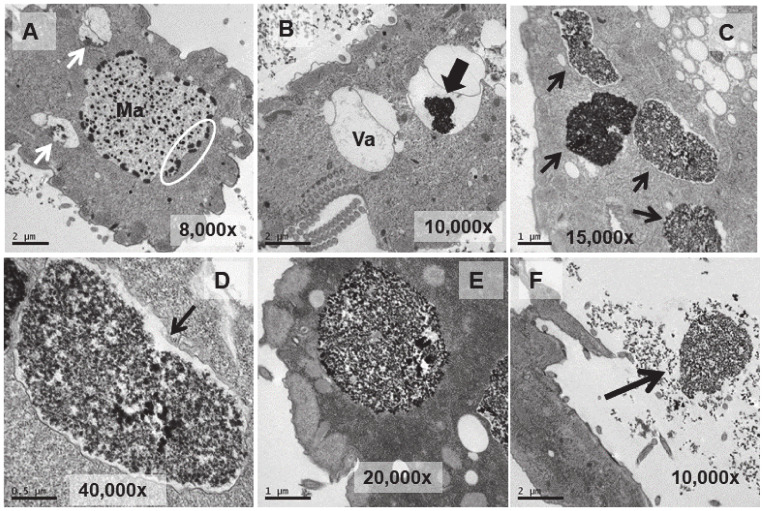
TEM images of cells treated with EuCl_3_ (2 mM, 1 h treatment). (**A**): Macronucleus (Ma) with a large number of nucleolar bodies (region within the ellipse). Vacuoles with an electrodense content (arrows). (**B**): Vacuole (Va). Black arrow indicates electrodense content. (**C**): Numerous vacuoles (arrows) containing an electrodense granular material (bioaccumulation). (**D**,**E**): Magnified images of vacuoles containing electrodense material. In (**D**) the vacuolar membrane is observed (arrow). (**F**): Ejection of the electrodense granular content (arrow) outside the cell.

Ultrastructural analysis was performed on cultures treated (1 or 24 h) with EuCl_3_, the two types of adapted strains (TtEuCl_3_-adap and TtEu_2_O_3_-adap) and a control culture (without any treatment). The analysis of Eu_2_O_3_-treated cultures was discarded due to of its low toxicity. Figure 4B–D show different cytoplasmic regions (macronucleus, vacuole, mitochondria) and an image of a whole *T. thermophila* cell (Figure 4A) as a control. Cells from a culture grown in PP210 and treated with EuCl_3_ (2 mM) for 1 h are shown in Figure 5. The macronucleus (Ma) is structurally similar to that shown by control cells, but with a greater number of nucleolar bodies (region within the ellipse) at its periphery (Figure 5A).

**Figure 6:**
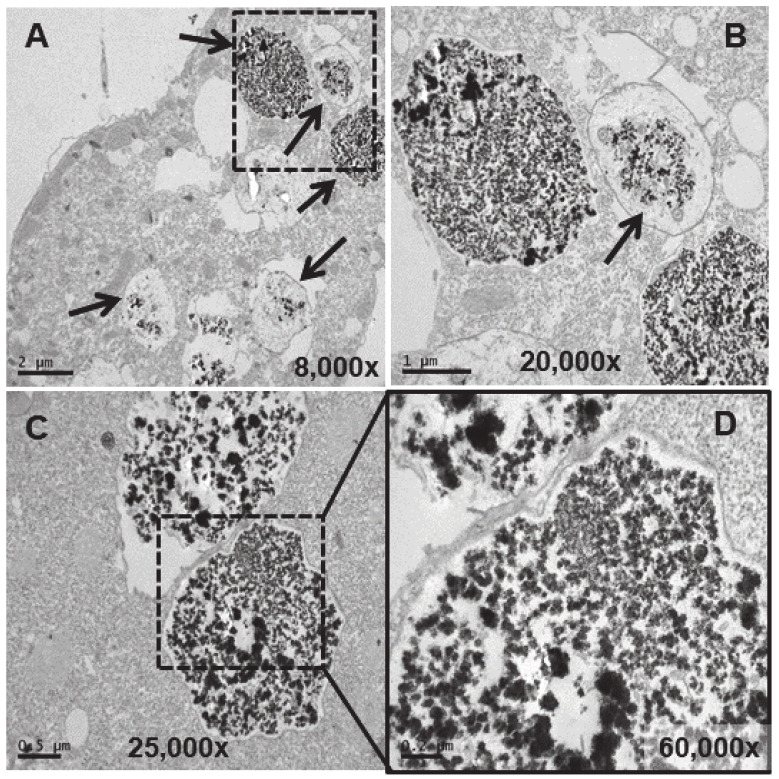
TEM images of cells treated with EuCl_3_ (2 mM, 24 h treatment). (**A**): Vacuoles with different levels of electrodense granular material bioaccumulation (arrows). (**B**): Magnification of the region (delimited by the square with the dashed line) in panel (**A**). (**C**): Vacuoles with granular electrodense material. (**D**): Magnification of the region (delimited by the square with the dashed line) in panel (**C**).

A larger number of vacuoles with an electrodense granular content are visible (arrows in Figure 5A,B). These vacuoles (~2–3 μm in length) bioaccumulate an electrodense granular material, presumably europium (Figure 5C–E). The vacuolar membrane enclosing this electrodense granular material is clearly visible in Figure 5D (arrow). Finally, this granular content is expelled from the cell by fusion of the vacuolar membrane with the ciliate envelope (Figure 5F). In the culture treated for 24 h with EuCl_3_, a higher number of vacuoles is observed at different bioaccumulation stages of the electrodense granular material (Figure 6A,B). The electrodense granules (~0.1 μm in diameter) condense into larger granules or clusters within the vacuole (Figure 6C,D).

**Figure 7:**
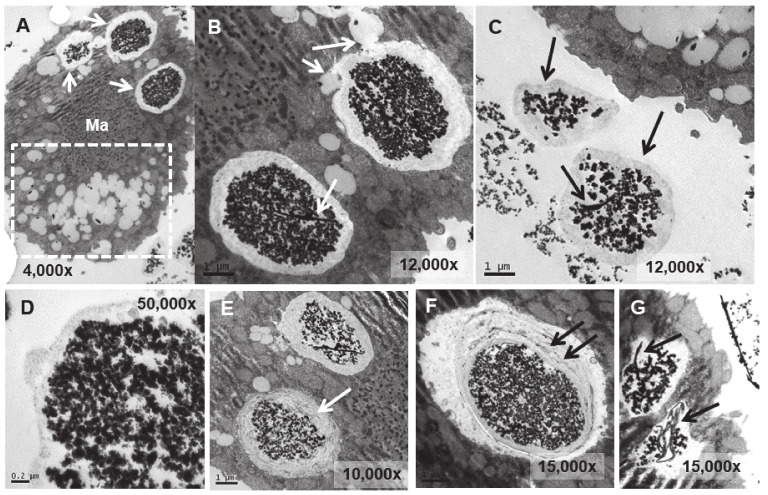
TEM images of TtEuCl_3_-adap cells. (**A**): Vacuoles with electrodense condensed granular content and surrounded by an electrolucent material originating from fusion with numerous lipid droplets (arrows). Region with numerous lipid droplets (dashed line box). Ma (macronucleus). (**B**): Magnified region from panel (**A**). (**C**): The granular contents from vacuoles (arrows) with their peripheral electrolucid region are excreted out of the cell. (**D**): Magnified detail of one of the excreted materials. (**E**,**F**): The electrolucent material sometimes contains membranous or fibrillar elements surrounding the electrodense granular content (arrows). (**G**): In some vacuoles, an electrodense fibrillar content is observed (arrows) together with the granular one.

In the TtEuCl_3_-adap strain, the electrodense granular content is more condensed within the vacuole and surrounded by a broad electrolucent region (Figure 7A,B,D). Membrane structures sometimes appear in this region surrounding the electrodense granular material (arrows in Figure 7E,F). In Figure 7A (dashed line box), a large number of lipid droplets are seen fusing with each other and with the vacuoles containing the electrodense granular material (arrows in Figure 7A,B). As the lipid droplet membrane fuses with that of the vacuole, it deposits its contents inside the vacuole, surrounding the granular material. When the granular contents of these vacuoles are excreted, they are also accompanied by the electrolucent envelope region (arrows in Figure 7C). The contents of some vacuoles also present electrodense fibrillar structures, in addition to the granular material, which are also expelled outside the cell (arrows in Figure 7C,G). 

**Figure 8:**
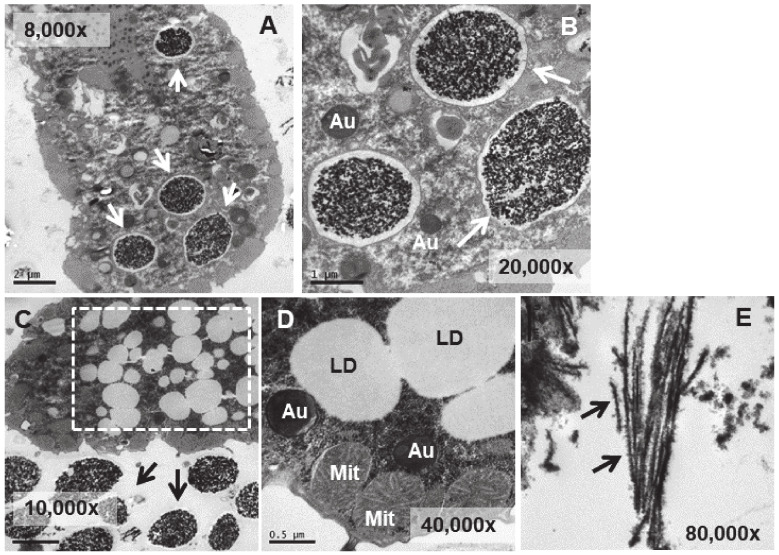
TEM images of TtEu_2_O_3_-adap cells. (**A**): Vacuoles with electrodense granular contents and a thin electrolucid envelope (arrows). (**B**): Magnified region from panel (**A**). Au (autophagosomes). (**C**): Region with numerous lipid droplets (dashed line box). Electrodense granular material ejected out of the cell (arrows). (**D**): Cytoplasmic region with mitochondria (Mit), autophagosomes (Au) and lipid droplets (LD). (**E**): Bundles of fibers with adhered electrodense material ejected out of the cell (arrows).

In the strain adapted to high concentrations of Eu_2_O_3_ (TtEu_2_O_3_-adap) the vacuoles containing the electrodense granular material are similar to those of the strain adapted to EuCl_3_ (TtEuCl_3_-adap), but the electrolucent envelope, deposited between the vacuolar membrane and the granular material, is thinner (Figure 8A,B). In addition, a large number of lipid droplets (dashed line box in Figure 8C) and numerous autophagosomes are detected in these cells. (Figure 8B,D). The electrodense granular content is expelled from the cell maintaining the shape it had inside the vacuole (Figure 8C), together with bundles of an electrodense fibrillar material (Figure 8E).

### 3.5. Microanalysis (TEM-XEDS)

**Figure 9:**
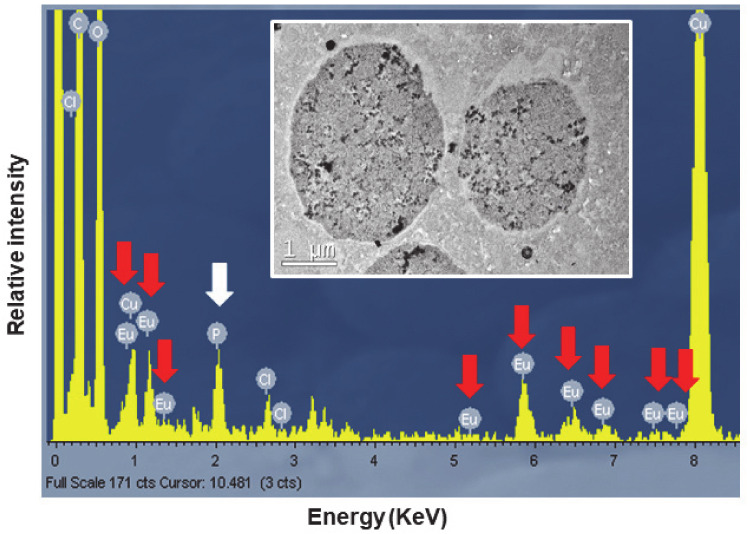
Spectra obtained by TEM-XEDS analysis. Internal TEM micrograph shows vacuoles on which elemental microanalysis was performed. Red arrows indicate the presence of europium, and the white arrow shows the presence of phosphorus.

To confirm the presence of europium, the TtEuCl_3_-adap strain was chosen for analysis of the elemental composition of the electrodense vacuolar deposits by TEM-XEDS. Figure 9 shows the spectrum obtained by TEM-XEDS from a semi-thin section of cells from the TtEuCl_3_-adap strain. An electron micrograph from which the microanalysis was performed is shown in Figure 9. The spectrum shows the characteristic peaks of europium (red arrows), as well as chlorine, carbon and oxygen. The presence of copper is due to the nature of the grid used. Phosphorus (white arrow) is also detected in the spectrum (Figure 9).

## 4. Discussion

As described in the introduction, europium compounds are of great interest in biology, medicine and various technologies related to imaging and optics. However, little is known about their potential toxicity in eukaryotic cells, and possible cellular strategies to counteract their toxicity such as bioaccumulation and/or biomineralization.

### 4.1. Toxicological and Growth Kinetics Parameters

As is the case with other metal(loid)s [50,51], the presence of organic matter in the medium significantly increases the value of the LC_50_ parameter with respect to that obtained in an inorganic medium (buffer), and this is due to the metal(loid)-chelating capacity of the organic matter [36]. Therefore, a higher amount of metal(loid) is required to obtain 50% cell mortality (LC_50_ value). The same is true for both europium compounds: in the PP210 growth medium with EuCl_3_ the LC_50_ value is increased by about 37-fold with respect to the value obtained in Tris-HCl, while with Eu_2_O_3_ this increase is about 45-fold. From the LC_50_ values it can be inferred that EuCl_3_ is more toxic (1.3–1.6-fold) than Eu_2_O_3_, which could be due to the higher solubility of EuCl_3_ in an aqueous medium, thus making Eu(III) more toxic.

The maximum concentration (5.5 mM) of EuCl_3_ used in the TtEuCl_3_-adap strain represents ~1.14-fold of the LC_50_ value of the wild-type (non-adapted) strain in PP210 or ~43-fold in Tris-HCl. In the TtEu_2_O_3_-adap strain, however, the maximum concentration achieved (8.5 mM Eu_2_O_3_) represents ~1.07-fold of the LC_50_ of the wild-type strain in PP210 or ~49-fold in Tris-HCl. 

In the ciliate *Paramecium bursaria* [31], 0.1 mM Eu(III) produces a mortality of almost 80%, a concentration much lower than the LC_50_ value obtained in *T. thermophila* for both europium compounds. This may be due to several factors, such as the europium compound used (europium acetate hydrate), the medium in which the treatment was performed, the cell concentration used and/or the ciliate species. 

The Eu(III) toxicity parameters used for other eukaryotic microorganisms are different. A 20% growth inhibition (IC_20_) of a yeast *Saccharomyces cerevisiae* cell population is achieved with EuCl_3_ 0.131 mM [52]. In the marine microalga *Skeletonema costatum* [53], 50% population growth inhibition or half-effective concentration (EC_50_) treated with europium nitrate is obtained at 29.16 μM. Regardless of the impossibility of comparing the toxicity values between different microorganisms, due to their very different parameters and conditions, we can conclude that *T. thermophila* seems to be more resistant to europium compounds than other eukaryotic microorganisms. 

Biotoxicity tests performed in distilled water on *T. thermophila* [54] with lanthanides other than Eu(III), such as La(III), Ce(III), Pr(III), Nd(III), and Gd(III), both in the form of oxides or nitrates, showed EC_50_ (24 h) values > 100 mg/L for rare earth oxides, and EC_50_ (24 h) values = 28–42 mg/L for nitrates. The authors suggest that these Ln concentrations are not sufficiently toxic for this ciliate. In another species, *T. shanghaiensis* [55], also using Ln other than Eu(III), the IC_50_ values (24 h and in rich growth medium) were between 0.34 mM (Gd) and 2 mM (La). 

The growth curve parameters of the two strains adapted to increasing concentrations of europium compounds show that the TtEuCl_3_-adap strain decreases its growth rate by about three-fold with respect to both the control strain and the TtEu_2_O_3_-adap strain (which has very similar growth parameters to the control), and the Tg increases by a factor of three. Consequently, this TtEuCl_3_-adap strain grows about three-fold slower than the control and the TtEu_2_O_3_-adap strain. This effect of reduced growth rate associated with metal adaptation was also found in other strains of this same ciliate adapted to Cd, Pb or Cu (unpublished data from our research group). 

In the thermophilic bacterium *Thermus scotoductus* [24] exposure to EuCl_3_ at low concentrations (0.01–0.5 mM) increases the maximum growth rate relative to the control, while at higher concentrations (1 mM) it decreases the growth rate. In contrast, in a *Clostridium* sp. strain [56], a decrease in growth rate is observed from a EuCl_3_ concentration as low as 0.01 mM, and the decrease is greater as the concentration increases. Therefore, under similar conditions the effect of the same Eu(III) compound can be very different depending on the microbial type. 

### 4.2. Oxidative Stress Assessment

None of the treatments performed with both Eu(III) compounds showed a significant increase over the untreated control. Therefore, we cannot assume that Eu(III) induces peroxide or hydroxyl radical generation in *T. thermophila*; which does not mean that other types of radicals that induce oxidative stress cannot be generated. However, in this same ciliate, exposure to oxides of several lanthanides other than Eu(III) induced oxidative stress (hydroxyl radicals) [54]. The main difference between these experiments and our results with Eu(III) is that they were performed in distilled water and not in growth medium, regardless of the lanthanides used. 

The non-detection of peroxide or hydroxyl radicals could be due to the protective system developed by the ciliate using antioxidant enzymes (such as catalase, glutathione peroxidase, peroxiredoxin reductase, thioredoxin reductase) to minimize the lethal effects of oxidative stress caused by Eu(III). 

Both Eu(OH)_3_ nano-bars and spheres, as well as Eu(NO_3_)_3_, induce angiogenesis (formation of new blood vessels, during embryonic development, growth and/or tumorization) in zebrafish embryos [57]. This induction of the angiogenic process is related to the production of H_2_O_2_ by these Eu compounds, i.e., Eu(III) → ROS (reactive oxygen species) → angiogenesis. In natural processes (embryogenesis), ROS production modulates angiogenesis through a reversible oxidase reaction [57]. Thus, there is a link between Eu(III) and the direct or indirect production of peroxides.

### 4.3. Expression Analysis of Genes Involved in General and/or Oxidative Stress Cell Response

For most of the genes analyzed, EuCl_3_ (2 mM, 24 h) and Eu_2_O_3_ (4 mM, 24 h) treatments are the ones that trigger their (sometimes significant) overexpression. Among the strains adapted to Eu(III) compounds, only the strain TtEu_2_O_3_-adap shows a significant (*p* ≤ 0.05) overexpression of the catalase-encoding gene (Figure 3E). This could corroborate the formation of a certain amount of peroxide radicals in this adapted strain, that catalase would degrade into water and oxygen. Although the DCF fluorescence results are not significant with respect to the control, a large standard deviation (SD) is observed, and according to the Brown–Forsythe test [58], the difference is significant at *p* < 0.05 (Figure 2). The adaptation of these strains means that many of the genes related to oxidative stress and those encoding metallothioneins, with the exception of catalase in the Eu_2_O_3_-adapted strain, do not need to be overexpressed.

The reduction of H_2_O_2_ to H_2_O involves the enzyme glutathione peroxidase and the reducing power is acquired from peroxiredoxins, and these acquire the reducing power from reduced thioredoxins, so thioredoxin reductases are important in this process. In the *T. thermophila* genome there are five thioredoxin reductase paralogous genes (*TrxR1*-*TrxR5*), of which three are selenoproteins and two (*TrxR2* and *TrxR5*) are not. We chose the latter two TrxRs isoforms because of their high overexpression with arsenic (arsenate) [42], a metalloid that causes increased oxidative stress. The results show that only the *TrxR5* isoform responds to both Eu(III) compounds, with a significant (*p* ≤ 0.01) overexpression obtained in the culture treated with Eu_2_O_3_. The same *TrxR5* gene from *T. thermophila* is overexpressed under treatment with the herbicide Paraquat that causes oxidative stress [34].

The overexpression (although not significant, probably due to the high values of their SDs) of the genes *GCL* (involved in glutathione (GSH) biosynthesis) and *GR1* (converts GSSG to GSH) could indicate the GSH requirement for glutathione peroxidases (GPx) which also reduce the H_2_O_2_ induced by Eu(III) treatments. Likewise, GSH is the substrate transferred by GSTs to potentially toxic molecules blocking their toxicity. Among the 70 GST paralogous genes present in the *T. thermophila* genome [59], only two (*GSTM3* and *GSTZ2*) have been selected for this study. A significant (*p* ≤ 0.05) overexpression of *GSTZ2* is obtained in the culture treated (24 h) with Eu_2_O_3_, and although there is also an induction of the *GSTM3* gene expression in both Eu(III)-treated cultures, these are not statistically significant (probably due to their large SDs). The *GSTZ2* gene is also overexpressed in *T. thermophila* with both selenite and selenate treatments, which cause increased oxidative stress [37]. 

Both superoxide dismutase genes (*CuZn-SOD* and *Fe-SOD*) are significantly induced against europium oxide (Figure 3D), especially *Fe-SOD* up to about 500-fold (*p* ≤ 0.0001). These enzymes convert the superoxide ion (highly toxic) to H_2_O_2_, which is then inactivated by catalase. Overexpression of both *SODs* would imply that superoxide ions are generated under europium oxide stress. Although both enzymes can be localized in the cytosol, Fe-SOD could also be located in the mitochondria [60], so the dramatic overexpression (Figure 3D) of this enzyme could also imply dysfunction in ciliate mitochondria. Another possible interpretation of the high increase in gene expression encoding Fe-SOD could be that SODs are ideal ligands for Eu(III) ions, as shown by the spectrofluorometric determination of these enzymes using an Eu-tetracycline probe [61]. If this interaction occurs in vivo it would block the enzyme, forcing the cell to synthesize much more of it. In various plants, other lanthanides (La, Ce) induce intracellular increases in SOD, CAT, GSH and the formation of hydroxyl ions, H_2_O_2_, superoxide ions and lipid peroxidation [2].

The ranking of the average relative induction values for EuCl_3_ (24 h) treatment is *GSTM3 > TrxR5 > Fe-SOD ≈ GR1 > GCL*, and for Eu_2_O_3_ (24 h) stress is *Fe-SOD >> TrxR5 > GSTM3 > GSTZ2 ≈ CuZn-SOD ≈ GR1 > GCL*. The first three antioxidant genes in both rankings coincide although in a different order and with very different induction values, as do the last two in the rankings (genes involved in glutathione metabolism). Although it is the same cation, Eu(III), it forms different compounds; one (EuCl_3_) with higher solubility and the other (Eu_2_O_3_) with nanoparticulate nature (45–58 nm) that is less water-soluble. These physical differences, as well as the type of ROS produced by the Eu(III) cation, could explain these differences in the two gene expression induction rankings.

Both europium compounds induce the expression of all *T. thermophila* MT genes, at different levels, except *MTT5*, which is only significantly induced (*p* ≤ 0.01) under europium oxide stress. In addition, the TtEu_2_O_3_-adap strain shows a significant (*p* ≤ 0.05) overexpression (2.6-fold) of the *MTT5* gene bordering the threshold of the minimum fold-change value (2-fold, dashed line in Figure 3A), which is considered by consensus as a significant relative quantification of the gene expression induction. Under EuCl_3_ stress (24 h), the ranking of *MTT* gene expression induction values is *MTT3 > MTT1 > MTT2/4*. However, under Eu_2_O_3_ stress (24 h), the ranking is *MTT1 >> MTT5 > MTT3 > MTT2/4*. As found in previous work [62,63], the ranking of these MT genes varies depending on the metal and treatment conditions. In this case, it is the same metal (Eu), although forming part of a different compound. 

In the study of the induction of *T. thermophila* metallothionein genes by metal(loid)s, it is common to use divalent cations [62], but it is more unusual to find studies with trivalent cations. Treatment (24 h) with arsenite [As(III)] induces overexpression of *MTT5* and *MTT1* genes (*MTT5 > MTT1*) [42], and lanthanum [La(III)] induces expression of *MTT1* and *MTT2* genes [64]. In the latter study, fluorescence analysis indicates that La(III) binds to both metallothioneins via the oxygen atoms of aspartic or glutamic acid residues. A fluorimetric method for quantification of MTs is based on the use of lomefloxacin-europium(III) complex as a fluorescent probe, since MT reacts with the LMLX-Eu(III) system to form a stable ternary complex (LMLX-Eu(III)-MT) [65]. Thus, in the case of both La(III) and Eu(III), it is shown that these trivalent cations can interact with these metal chelating proteins. Furthermore, a toxigenomic analysis [52] using EuCl_3_ suggests that Eu(III) can disrupt the function of chaperones and cochaperones that present metal binding sites, thus promoting toxicity in yeast. This could also explain the overexpression of the *MTT3* gene by EuCl_3_ treatment, which, together with *MTT1*, is one of the genes with the highest basal constitutive expression, and is thought to play a role in the intracellular homeostasis of essential metals such as Zn(II) or Cu(II) [63]. Since it may be disrupted by Eu(III), the cell requires more of it for its viability. Lanthanides react with biologically active compounds that replace Ca(II) ions with, among others, Zn(II), Mg(II), Fe(II) [66]. If Zn(II) in MTT3 is replaced by Eu(III) blocking the function of this MT, the cell would need to synthesize more of this protein, hence the increased overexpression of this *MTT3* gene.

### 4.4. Ultrastructural Modifications and Microanalysis

Ultrastructural analysis of the culture treated with EuCl_3_ (1 h) shows an increase in the number of nucleolar bodies in the macronucleus of *T. thermophila*. It is known that nucleoli undergo structural changes as a cellular response to many environmental stressors (known as “nucleolar stress”), thus serving as a bioindicator of the cellular stress [67]. An increase in the number of nucleoli could imply a greater need for ribosome biosynthesis to keep the cell growing despite the toxic effect of europium on cell growth (as in the TtEuCl_3_-adap strain, where its growth rate decreases about three-fold compared to the control). The increase in the number and size of nucleoli has been used as an indicator of cancerous lesions in many types of tumors, and this increase is attributed to the need for protein biosynthesis in cancer cells [68,69]. Similarly, in hypertrophied human hearts (with hyperfunction) the number of nucleoli is increased, indicating an increase in RNA synthesis [70].

Another characteristic of these cells is an increase in the number of vacuoles with a granular electrodense content. This material (europium) is eventually released from the cell. In longer treatments (24 h) with EuCl_3_, the number of vacuoles increases, and different bioaccumulation phases of this material inside the vacuoles are observed. In the literature on lanthanide bioaccumulation (including europium), the most quantitatively relevant is that carried out by microorganisms (non-photosynthetic) and phytoplankton (including microalgae) [1]. Other authors [71] also highlight the Ln bioaccumulation by zooplankton, as an excellent bioindicator of their bioavailability in freshwater ecosystems. This process of Eu(III) bioaccumulation, probably complexed with biomolecules, and subsequent elimination outside the cell, represents a detoxification mechanism (widespread among eukaryotes) that involves an increase in vacuolar activity. 

The main structural difference between the electrodense granule-containing vacuoles from the EuCl_3_-treated culture and the TtEuCl_3_-adap or TtEu_2_O_3_-adap strains is the thick electrolucid region surrounding the bioaccumulated material. This electrolucid region is formed by fusion of the vacuolar membrane with numerous lipid droplets. Some of these regions contain membranous debris to which europium can bind and form electrodense fibrillar structures. 

Lipid droplets can be biomarkers, vehicles, and facilitators for cellular stress response and survival [72]. Lipid droplets, as potential sources of nutrients and energy, respond to starvation stress [72]; they are associated with autophagy [73], they are involved in cross- organelle communication [74], and are found in infectious diseases due to viruses, bacteria or protozoa [75]. Indeed, in the TtEu_2_O_3_-adap strain, numerous autophagosomes are detected together with large lipid droplets (Figure 8D), indicating that both processes are related to stress caused by europium oxide nanoparticles. Similarly, an increase in lipid droplets has been observed in various eukaryotic cells under metal(loid) stress, such as Cd(II) [76] or Cu(II) [77], and in *T. thermophila* under metal nanoparticle stress (copper oxide nanotubes) [78] or As(III) treatment [42]. 

When pathogenic microorganisms infect a eukaryotic cell, e.g., *Chlamydia* bacteria or the protozoan parasite *Toxoplasma*, lipids appear to accumulate due to the trafficking of lipid droplets from the host cell to the vacuoles where the pathogen replicates [72]. It is possible that a similar mechanism of isolation of toxic particulate elements (such as europium nanoparticle aggregates) could occur in *T. thermophila* cells under the extreme stress experienced by strains adapted to high concentrations of Eu(III) compounds. Once the toxic element is isolated in a vacuole with membrane remnants and high lipid content, it would be expelled from the cell. 

TEM-XEDS microanalysis of the electrodense granular content of the vacuoles of the TtEuCl_3_-adap strain showed a spectrum with the 8–9 peaks or regions where Eu(III) is detected, very similar to that shown by other authors [24]. This confirms that the vacuolar content in these cells contains europium. In addition, a significant peak identified as phosphorus appears in the same spectrum. The soluble Eu(III) could react with cytoplasmic phosphates or polyphosphates to form europium phosphate (EuPO_4_). 

Both intracellular and extracellular (biosorption) nano-biomineralization, and bioaccumulation of lanthanides (including europium) have been described in both bacteria [24,79] and eukaryotic microorganisms (yeast and microalgae) [79,80]. The thermophilic bacterium *T. scotoductus* [24] can bioaccumulate intra- and extra-cellular Eu(III), which is biomineralized as Eu_2_(CO_3_)_3_ (europium carbonate). Likewise, Eu(III) intracellular bioaccumulation could be facilitated by polyphosphate metabolism. In fact, both electron microscopy and microanalysis have shown intracytoplasmic electrodense granules composed of Eu(III) and phosphate [24]. Lanthanide phosphates and carbonates are insoluble under physiological conditions and therefore precipitate. Both in vivo and in vitro, using the microalga *Chlorella vulgaris* [80], Eu(III) chloride binds preferentially to phosphate groups. 

Several studies have reported phosphate mineralization of both light (Ce) and heavy (Yb) lanthanides in the yeast *S. cerevisiae*. Needle-shaped Ce(III) phosphate nanocrystals were detected in *S. cerevisiae* cells after exposure to a Ce(III) solution [81]. Similarly, the formation of ytterbium (Yb) phosphate nano-particles on the cell surface as a precipitate after an adsorption process, has been described in the same yeast [82]. In a strain of *T. thermophila* adapted to high levels of Pb(II), a process of biomineralization of this metal to chloropyromorphite (Pb_5_[PO_4_]_3_Cl) based on the utilization of intracellular phosphate, has been studied [83]. It is therefore not surprising that, as in other microorganisms, phosphate is used to carry out a detoxification process. 

## 5. Conclusions

In *T. thermophila*, EuCl_3_ is more toxic than Eu_2_O_3_. Nevertheless, this microorganism seems to be more resistant to europium compounds than has been reported for other eukaryotic microorganisms. 

Cell adaptation to EuCl_3_ affects the growth rate of the adapted strain, but does not affect the growth of the Eu_2_O_3_-adapted strain.

The absence of peroxides or hydroxyl radicals after treatment with both Eu(III) compounds could be due to the protective system developed by the ciliate, with the intracellular increase of antioxidant enzymes, as partially confirmed by the overexpression of the genes encoding them. 

The overexpression of metallothioneins under treatment with Eu(III) compounds supports the possibility that this lanthanide may interact with the -SH groups of the cysteine residues of MTs and/or displace essential cations of these proteins during their homeostatic function.

Both lipid metabolism and autophagy are involved in the cellular response to europium stress.

As in other microorganisms, the main detoxification mechanism of Eu(III) compounds in *T. thermophila* is bioaccumulation in vacuoles and subsequent efflux from the cell, probably linked with a biomineralization process to europium phosphate.

## Figures and Tables

**Table 1:** LC_50_ (μM) values obtained from the dose–mortality curves shown in Figure S1.

Medium	EuCl_3_ (μM)	Eu_2_O_3_ (μM)
Tris-HCl	127.93	173.32
PP210	4830.68	7916.10

## Data Availability

Data will be made available on request.

## Supplementary Materials

The following supporting information can be downloaded at: https://www.mdpi.com/article/10.3390/biology13050285/s1, Figure S1: Dose–mortality curves. (**A**): EuCl_3_ treatments. (**B**): Eu_2_O_3_ treatments. Histograms: (A1, A3, B1 and B3). Adjusted model with 95% confidence intervals: (A2, A4, B2 and B4); Table S1: Sequences and features of the primers used in the qRT-PCR analysis; Table S2: Quantitative RT-PCR standard-curve parameters.
