# Supplementary material for: Cellular Response of Adapted and Non-Adapted Tetrahymena thermophila Strains to Europium Eu(III) Compounds
Source: Biology (Basel). 2024 Apr 23;13(5):285. doi: 10.3390/biology13050285 (PMC11117543; doi:10.3390/biology13050285)

Supplementary material

Table S1. Sequences and features of the primers used in the qRT-PCR analysis.

Primers[(1)]	Length (b)	%(G+C)	Tm (ºC)	Sequences (5 --> 3')
TtACT1	22	40.9	52.8	CTC TCT TTC TAC CTT CCA AAC T
TtACT2	21	38.1	52.1	AGG ACC AGA TTC ATC ATA TTC
TtCATA	21	47.62	54.9	GCT TTC TCT CCT TCC CAC TTA
TtCATB	21	47.62	54.3	TCT GTG GGT ATC GGG ATA AGA
TtGR1A	16	43.75	36	ACT TAG ACA GGT GAA C
TtGR1B	16	50	38	ACT CAA GGT TAA CGC C
TtGCLA	24	33.33	56	AGA ATT CAG AAC TAT GGA ATG CTA
TtGCLB	20	45	57	ATG AAA GCA GCG TTC TCA TC
MTT1QA	26	34.6	53.6	ATG GAT AAA GTT AAT AGC TGT TGC TG
MTT1QB	17	47.1	49.6	AAA GCA GCA GGG TTT AG
MTT2/4QA	17	41.2	46.7	ATG CAA ATG TGG ATC TC
MTT2/4QB	17	47.1	46.6	CAG TTG GAA GTA GAA CC
MTT3QA	17	29.4	43.4	ACA ATG TAA TTG TGC TT
MTT3QB	17	41.2	48	TAA GCA GCT GGA TTT GA
MTT5QA	21	52.4	57.8	TGT GTC GGT TCA GGA GAA GGA
MTT5QB	23	43.5	57	AGG TCC GCA TTT ACA TTC AGC TT
TtSODCuA	23	43.48	54.4	GAT GAT TTA GGT AGA GGC AAC CA
TtSODCuB	22	40.91	54.3	AGC AAG TCC AAT AAT ACC AGC A
TtSODFeA	22	45.45	54.3	CCT CAC CAA GGC CAT ATA AGA A
TtSODFeB	20	45	54.9	AGT GTC ATA ACL GAG CCA AC
TtGSTM3A	20	50	47	CTA TAG GAG CTG GGA TCA CT
TtGSTM3A	19	42.11	42	GAA AAA GCA CCA TGA TAC C
TtGSTZ2A	19	42.11	42	CAG TGC TAT TCA TCC CTA T
TtGSTZ2B	19	31.58	37	TCA TAT TGC CTT CTT TTT C
TtTR2A	19	26.67	44	CTA AGT AAG TAG TT A
TtTR2B	20	26.32	45	TAT CTT TAT CTG ATA GTA C
TtTR5A	21	38.1	52	TTT GCA ATA GGA GAT GCT GTT
TtTR5B	21	42.86	52	CCC TTA TTT TAT TGC CAC AGG

[(1)] TtACT1/2: Beta-actin, TtCATA/B: Catalase, TtGR1A/B: glutathione reductase, TtGCLA/B: glutathione cysteinyl ligase, MTT1QA/B: MTT1 metallothionein, MTT2/4QA/B: MTT2/MTT4 metallothioneins, MTT3QA/B: MTT3 metallothionein, MTT5QA/B: MTT5 metalothionein. TtSODCuA/B: CuZn-superoxide dismutase, TtSODFeA/B: Fe-superoxide dismutase, TtGSTM3A/B: M3 glutathione transferase, TtGSTZ2A/B: Z2 glutathione transferase, TtTR2A/B: thioredoxin reductase 2, TtTR5A/B: thioredoxin reductase 5.

Table S2. Quantitative RT-PCR standard-curve parameters.

Gene	S[1]	E (%)[1]	Y-intercept	R^{2} [1]
β-actin	-3.266	102.30	16.714	0.997
MTT1	-3.068	111.80	16.304	0.998
MTT2/4	-3.123	108.99	17.953	0.998
MTT3	-3.506	92.85	10.022	0.992
MTT5	-3.602	89.51	7.897	0.955
GCL	-3.252	103.00	15.123	0.998
GSTM3	-3.667	87.37	18.171	0.991
GSTZ2	-3.861	81.55	19.725	0.996
CAT	-3.168	106.81	20.01	0.989
CuZn-SOD	-3.235	103.74	15.59	0.991
Fe-SOD	-3.392	97.12	11.419	0.998
TRX5	-3.722	85.64	19.807	0.983
TRX2	-3.517	92.43	16.797	0.977
GR1	-3.209	104.92	18.722	0.988

[1] Slope (S), amplification efficiency (E), correlation coefficient (R^2).

Figure S1. Dose-mortality curves. (A): $EuCl_3$ treatments. (B): Eu_2O_3 treatments. Histograms: (A1, A3, B1 and B3). Adjusted model with 95% confidence intervals: (A2, A4, B2 and B4)

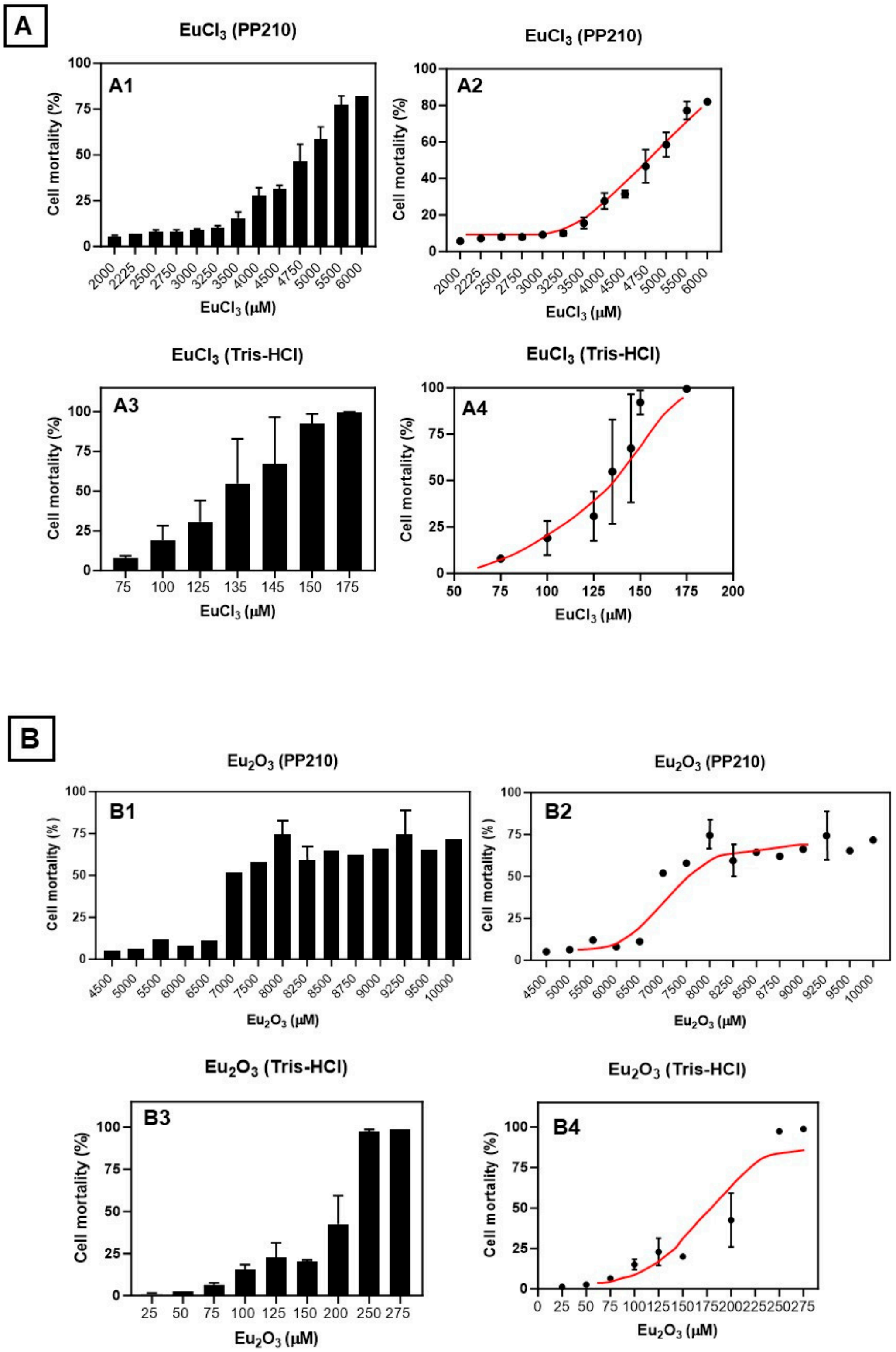

Supplement: Supplementary file 1 [file biology-13-00285-s001.zip › biology-2946120-supplementary.pdf]
